# Epidemiological and viral features of a cohort of SARS-CoV-2 symptomatic and asymptomatic individuals in an area of the Colombian Caribbean

**DOI:** 10.1186/s12941-020-00397-5

**Published:** 2020-12-07

**Authors:** Salim Mattar, Caty Martinez-Bravo, Ricardo Rivero, Héctor Contreras, Álvaro A. Faccini-Martínez, Camilo Guzman-Teran, Ketty Galeano, Nelson Alvis-Guzman, Verónica Contreras, German Arrieta, Marco Gonzalez-Tous, Jorge Miranda, Martha Ospina, Francisco Camargo-Assis, Marcela Mercado-Reyes, Evelyn Garay, Alejandra Garcia-Perez, Yesica Lopez, Vaneza Tique

**Affiliations:** 1 Instituto de Investigaciones Biológicas del Trópico, Facultad de Medicina Veterinaria y Zootecnia, Universidad de Córdoba, Montería, Colombia; 2 Asociación Colombiana de Infectología, Carrera 15 No. 118-03, Bogotá, Colombia; 3 University of Cartagena, ALZAK Foundation, Cartagena, Colombia; 4 Instituto Nacional de Salud, Bogotá, Colombia; 5 Intensive Care Unite, Clinica Zayma, Montería, Córdoba Colombia

**Keywords:** Asymptomatic infections, COVID-19, Coronavirus infections, Pre symptomatic disease, Environment and public health, Communicable disease control

## Abstract

**Background:**

Severe acute respiratory syndrome Coronavirus 2 (SARS-CoV-2) is an emerging viral pandemic disease. In the last 6 months, SARS-CoV-2 has caused millions of reported cases and hundreds of thousands of deaths. As other world regions, South America has not contained the pandemic’s advance since it lacks the hospital and economic capacities. Public health implications of transmission, while the asymptomatic/presymptomatic infection is a critical concern at the current pandemic.

**Objective:**

Describe the socio-demographic, clinical, and viral features of a cohort of SARS-CoV-2 infected individuals from the Colombian Caribbean.

**Methods:**

Six hundred eighty-six clinical samples of suspected SARS-CoV-2 infection cases and contacts individuals from several hospital centers in the department of Córdoba, Colombia, were received at our laboratory between April 9th and May 16th, 2020. RNA was extracted using lysis buffers and spin columns. The samples were tested for SARS-CoV-2 by reverse transcription real-time polymerase chain reaction (RT-qPCR) using commercially available multiplex real-time PCR assay for simultaneous detection of 3 target genes of SARS-CoV-2 (Allplex™, 2019-nCoV assay, Korea). Viral copies quantification was done using a standard curve constructed from seriated dilutions of a SARS-CoV-2 positive control. Statics descriptive methods were used.

**Results:**

Thirty-five nasopharyngeal samples were positive for SARS-CoV-2 infection; the average age was 43 (range, 1–95 years). Seventeen of 35 (49%) of the patients showed symptoms. Most of them had a cough, fever, and odynophagia; three of the patients reported having arthralgia. Only two patients required hospitalization. None of the patients had known co-morbidities. RT-qPCR results show that two of the symptomatic patients had significantly higher RNA copies than the rest. Eighteen of 35 (51%) individuals were asymptomatic, and the average age was 30 (range, 6–61 years). Four asymptomatic individuals showed a higher copy than some symptomatic patients; nonetheless, the average of RNA copies 8.26 × 10^10^ was lower than the symptomatic.

**Conclusions:**

This study shows that asymptomatic patients may develop infections with a high number of RNA copies. Since a considerable percentage of infections may be asymptomatic/presymptomatic, enhanced testing approaches may be needed to detect these persons. Due the occurrence of a large proportion of infections being a result from transmission originated in asymptomatic/presymptomatic individuals, public health interventions in Colombia should be based on two steps: a massive molecular screening, and viral load quantification. Finally, a remarkable issue in our study is the average age of symptomatic and asymptomatic groups (43 and 30 respectively) which may be important because of the economic impact that has been caused by the coronavirus pandemic and may be probably the cause of the reduced lethality observed in the country and the department at the time of this study.

## Introduction

Coronavirus disease 2019 (COVID-19), caused by Severe acute respiratory syndrome Coronavirus 2 (SARS-CoV-2), is an emerging viral disease that has caused millions of reported cases and hundreds of thousands of deaths in the last 6 months. As other world regions, nowadays, South America has not contained the pandemic’s advance since it lacks the hospital and economic capacities, reporting more than nine million of infected people, being the top six countries’ distribution as follows: Brazil has 5,224,362 cases, Argentina 979,119, Colombia 952,371, Peru 865,549, Chile 490,003, and Ecuador 152,422 cases [1]. Besides, the mortality per million people in Peru, Brazil, Bolivia, Chile, Ecuador, Argentina, and Colombia are 1.018, 722, 722, 709, 698, 576, and 564, respectively, with a total of 276,725 people who died (99.25% of the total deaths in South America) [1]. In Colombia, the first case of SARS-CoV-2 infection was reported on March 6th, 2020, and has been shocked by the national incidence rate of 1,834.5 cases per 100,000 inhabitants [2, 3]. There have been 28,306 deaths from COVID-19 in the country, of which 64.2% correspond to men. 75.8% of the deceased are in the age group 60 and over [3]. The people with the highest number of deaths are between 70 and 79 years old, with 7321, followed by the group between 60 and 69 years old, with 6542 deaths [3].

Cordoba department, in the Colombia Caribbean area, reported its first case on March 26th. On October 16th, 2.65% of cases did require hospitalization. However, the mortality rate reached 869.4/million, higher than the national rate of 575.1/million [2]. Implications in public health of pathogen transmission by patients with asymptomatic infections are a critical concern at the current pandemic [5, 9].

This study aims to describe the socio-demographic, clinical, and viral features of a cohort of SARS-CoV-2 infected individuals from the Colombian Caribbean.

## Methods

### Type of study, geographic localization, and sample collection

**Fig. 1:**
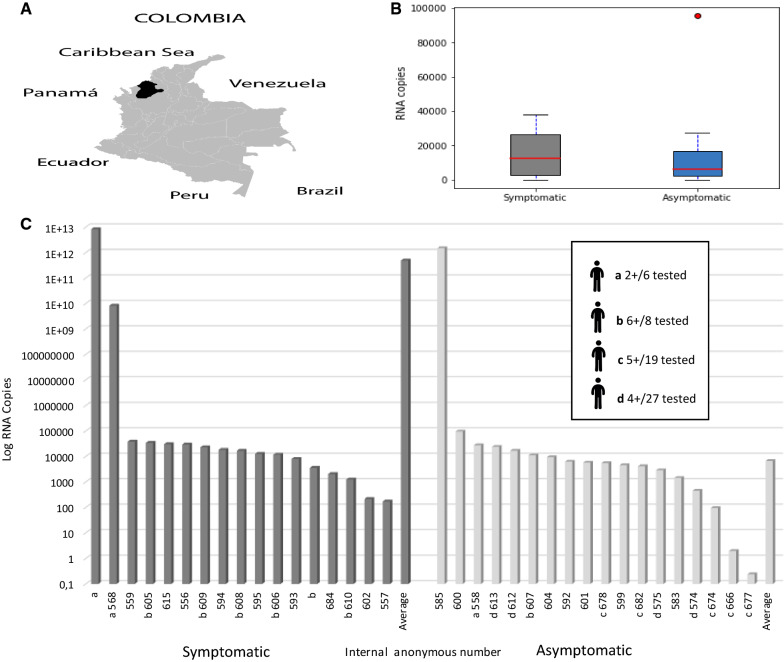
**A** Map of Colombia with its neighborhood countries. **B** Boxes of the median of RNA copies and bars of individual symptomatic patients and asymptomatic subjects with RNA copies. Asymptomatic data. Median = 6003.985; P25 = 2152.519; P75 = 16,780.655. Symptomatic data: median = 12,652.686; P25 = 2813.048; P75 = 26,288.189. Extreme values were excluded, red spot outlayer extreme. The median RNA copies for symptomatic was 12,652.6 (IQR 2813.0–26,288.1) and for asymptomatic 6003.9 (IQR 2152.5–16,780.6). **C** X-axis internal patient’s code number, Y-axis Log by RNA copies/ml of symptomatic and asymptomatic individuals, the average of symptomatic was higher than asymptomatic patients. Several asymptomatic patients show higher RNA copies than some symptomatic patients. Confirmed patients a and b resulted in 2 and 6 infected contacts, respectively. Asymptomatic individuals c and d resulted in 5 and 4 asymptomatic individuals, some of them with important RNA copies. The average of symptomatic was higher than asymptomatic. A remarkable data that 15 health workers resulted infected in order according to viral load (symptomatic = 556, 593, 594, 595, 602; asymptomatic = 583, 585, 592, 674, 677, 678, 682, 600, 601 and 604)

The present work is a prospective, descriptive study carried out in Cordoba, located in the northwest of Colombia; it is part of the Caribbean savanna (Fig. 1A). The mean annual temperature is 28 °C, prevailing a dry and a rainy season. The department’s population is 1,828,947 inhabitants, and Monteria, Cordoba’s capital city, has a population of 505,334. This study was conducted at Instituto de Investigaciones Biológicas del Trópico, Universidad de Cordoba, which is licensed by Colombia's National Health Institute for the molecular diagnostic of SARS-CoV-2 human infection. Six hundred eighty-six clinical samples of suspected SARS-CoV-2 infection cases and contacts individuals from several hospital centers in the province were received and processed by RT-qPCR between April 9th and May 16th, 2020, with 35 positive results for SARS-CoV-2 infection. The ethical standards of the Ministry of Health of Colombia Resolution No. 8430 of 1993 were followed. The data of the present study correspond to patients coded under strict anonymity with an internal laboratory number.

### RNA extraction and SARS-CoV-2 detection

Briefly, RNA was extracted using lysis buffers and spin columns. After RNA extraction, the samples were tested for SARS-CoV-2 by RT-qPCR using commercially available multiplex real-time PCR assay for simultaneous detection of 3 target genes of SARS-CoV-2 (AllplexTM, 2019-nCoV assay, Korea). The test is designed to detect RNA dependent RNA polymerase (RdRP), N genes specific for SARS-CoV-2, and E gene for all of Sarbecoviruses, including SARS-CoV-2. Viral copies quantification was done using a standard curve constructed from seriated dilutions of a SARS-CoV-2 positive control. This control includes synthetic RNA target sequences for the three genes (RdRP, S, and N). According to the manufacturer instructions, samples tested with a Ct value ≤ 40 were considered positive [6, 7]. If a tested sample had a Ct between 40 and 45, the test was repeated.

## Results and discussion

Presently, the cases of asymptomatic/presymptomatic people represent the pandemic’s significant problem due to its great spreading power of the coronavirus, which makes the control of the disease much more difficult. It is like fighting an invincible enemy; thus, it is vital to study healthy people. In that sense, while Colombia has tested 86,232 per million people in a country with 50 million people, Brazil, with 213 million people, has tested 84,035 individuals, and Peru and Chile are the countries with more tested people in South America, exceeding both the 328,353 tests [1].

In the present study, 35% of nasopharyngeal samples were positive for SARS-CoV-2 infection. The average age was 43 (range, 1–95 years). Seventeen of 35 (49%) of the patients showed symptoms, 9/17 (53%) of them were female, eight patients were from the urban area of Monteria (505,334 inhabitants), eight from Sahagun (107,636 inhabitants), and one from Cerete (105,815 inhabitants). Moreover, most of them showed cough, fever, and odynophagia; 3 of the patients reported having arthralgia (Table 1). Only two patients required hospitalization. So far, all of the symptomatic patients are alive and have recovered from the infection. Both pediatric patients were symptomatic (Table 1). None of the patients had known co-morbidities. Only four symptomatic patients reported not having expositional contact with an infected person. RT-qPCR results show that two of the symptomatic patients (codes 505, 568) had significantly higher RNA copies than the rest of them. The average RNA copies were very high 4 × 9^11^ (Table 1). Asymptomatic individuals 585, 600, 613, and 612 showed a higher number of RNA copies than some symptomatic patients (Table 1, Fig. 1C). Nonetheless, the average of RNA copies 8.26 × 10^10^ was lower than the average of the symptomatic group. The Wilcoxon test for independent samples showed that there is no significant difference (p > 0.05) between the viral RNA copy number of symptomatic and asymptomatic patients (Fig. 1B). Nine of 17 individuals were from Monteria, the remaining nine from Sahagun. Forty-three of the health staff were infected, an important issue because they are in the front line facing the pandemic (Table 1).

**Table 1:** Epidemiological, clinical, demographic information, and RNA virus copies

Anonymous code	Date of sample	Symptomatic (onset date)/asymptomatic	Municipality	Age/gender/health staff	Contact	Ct	RNA copies/ml
505a	9/05/2020	Symptomatic (30-Apr) Fe, Od	Sahagun	65/M/no	No	24.00	8.33E+12
556	11/05/2020	Symptomatic (9-May) Co, Ar, Od	Sahagun	32/F/yes	Yes	36.94	2.96E+04
557	12/05/2020	Symptomatic (10-May) Fe, Ar, Od	Sahagun	95/M/no	No	39.27	1.70E+02
568a	12/05/2020	Symptomatic (9-May) Od	Sahagun	52/M/no	Yes	24.03	8.26E+09
593	13/05/2020	Symptomatic (6-May) Co, Od	Sahagun	28/F/yes	Yes	38.64	7.97E+03
594	13/05/2020	Symptomatic (11-May) Co, Od	Sahagun	31/F/yes	Yes	37.42	1,82E+04
595	13/05/2020	Symptomatic (11-May) Co	Sahagun	56/M/yes	Yes	38.04	1.27E+04
615	13/05/2020	Symptomatic (10-May) Fe	Sahagun	24/F/no	Yes	36.91	3.05E+04
517b	9/05/2020	Symptomatic (2-May) Co, Od	Monteria	26/M/no	No	37.12	3.59E+03
602	12/05/2020	Symptomatic (10-May) Co, Od	Monteria	41/F/yes	Yes	40.37	2.15E+02
605b	13/05/2020	Symptomatic (4-May) Co	Monteria	23/F/no	Yes	34.71	3.43E+04
606b	13/05/2020	Symptomatic (10-May) Co	Monteria	60/M/no	Yes	38.13	1.18E+04
608b	13/05/2020	Symptomatic (11-May) Co	Monteria	43/F/no	Yes	37.67	1.68E+04
609b	13/05/2020	Symptomatic (10-May) Od	Monteria	1/M/no	Yes	37.27	2.30E+04
610b	13/05/2020	Symptomatic (12-May) Fe	Monteria	6/M/no	Yes	41.18	1.24E+03
684	15/05/2020	Symptomatic (1-May) Ar	Monteria	72/F/no	Yes	37.75	2.04E+03
559	11/05/2020	Symptomatic (7-May) Od	Cerete	74/F/no	No	36.62	3.82E+04
558a	11/05/2020	Asymptomatic	Sahagun	46/F/no	Yes	36.62	2.76E+04
583	13/05/2020	Asymptomatic	Sahagun	32/F/yes	Yes	37.63	1.43E+03
585	13/05/2020	Asymptomatic	Sahagun	30/F/yes	Yes	24.84	1.49E+12
592	13/05/2020	Asymptomatic	Sahagun	21/M/yes	Yes	38.96	6.26E+03
666c	15/05/2020	Asymptomatic	Sahagun	34/F/no	Yes	42.94	1.93E+00
674c	15/05/2020	Asymptomatic	Sahagun	27/F/yes	Yes	39.73	9.41E+01
678c	14/05/2020	Asymptomatic	Sahagun	30/F/yes	Yes	36.63	5.49E+03
682c	15/05/2020	Asymptomatic	Sahagun	50/M/yes	Yes	36.84	4.12E+03
574d	12/05/2020	Asymptomatic	Monteria	26/M/no	Yes	38.51	4.52E+02
575d	12/05/2020	Asymptomatic	Monteria	17/F/no	Yes	37.11	2.87E+03
599	13/05/2020	Asymptomatic	Monteria	35/M/no	Yes	39.39	4.57E+03
600	12/05/2020	Asymptomatic	Monteria	26/M/yes	Yes	33.66	9.54E+04
601	12/05/2020	Asymptomatic	Monteria	25/M/yes	Yes	39.08	5.75E+03
604	12/05/2020	Asymptomatic	Monteria	61/M/yes	Yes	36.08	9.34E+03
607d	13/05/2020	Asymptomatic	Monteria	17/M/no	Yes	38.21	1.11E+04
612d	13/05/2020	Asymptomatic	Monteria	24/F/no	Yes	37.67	1.68E+04
613d	13/05/2020	Asymptomatic	Monteria	6/F/no	Yes	35.57	2.38E+04

*Fe* fever, *Co* cough, *Ar* arthralgia, *Od* odynophagia, *M* male, *F* female

On the other hand, 18/35 (51%) individuals were asymptomatic. All of them had a known infected contact (Table 1); the figure shows the number of infected contacts (Fig. 1C). We do not know whether asymptomatic individuals in the present study developed COVID-19 disease after taking the sample. Of the asymptomatic individuals, 10/18 (56%) were female, the average age was 30 (range, 6–61 years. Most studies report that males are more affected by coronaviruses than females; however, in the present study, women were more affected than men (53% symptomatic and 56% asymptomatic). This trend, 52.31%, continues throughout Cordoba’s department and is opposed to national behavior (49.5%) [2]. Our study has some weaknesses, such as a small sample of individuals and a non-follow-up change from presymptomatic to symptomatic. However, the present study reinforces the concern about the public health implications of asymptomatic/presymptomatic SARS-CoV-2 infection [3–8]. Our results show a 51% of asymptomatic infected individuals, of which 78% (14/18) presented a considerably high viral copy number, even higher than several symptomatic patients (Fig. 1C). Moreover, RT-PCR Ct values lower than 34 of some them indicate a higher viral load than some symptomatic patients and presumes the possibility to isolate infectious SARS-CoV-2 to demonstrate viral viability [3, 6, 8, 9].

## Conclusion

Since a considerable percentage of infections may be asymptomatic, increased testing approaches may be needed to detect these persons [10, 11]. Because a large proportion of infections may result from transmission originated in asymptomatic or pre-symptomatic persons, the usefulness of public health interventions in Colombian departments should be based on two steps: a molecular screening in a vast conglomerate’s population, and viral load quantification. Finally, a remarkable issue in our study is the fact of young age in symptomatic and asymptomatic individuals (average of 43 years and 30 years, respectively); age group data is essential because the coronavirus pandemic has produced a high impact in the economy and it is probably the cause of the reduced lethality observed in the country and the department at the time of this study.

## Data Availability

Our results are preliminary, and we do not wish to share this preliminary data for the moment.
